# Bovine Papillomavirus Type 2 Infection and Microscopic Patterns of Urothelial Tumors of the Urinary Bladder in Water Buffaloes

**DOI:** 10.1155/2013/937918

**Published:** 2013-05-22

**Authors:** Paola Maiolino, Ayhan Özkul, Aylin Sepici-Dincel, Franco Roperto, Gözde Yücel, Valeria Russo, Chiara Urraro, Roberta Lucà, Marita Georgia Riccardi, Manuela Martano, Giuseppe Borzacchiello, Iolanda Esposito, Sante Roperto

**Affiliations:** ^1^Department of Veterinary Medicine and Animal Productions, Naples University Federico II, Via Delpino 1, 80137 Naples, Italy; ^2^Department of Pathology, Faculty of Veterinary Medicine, Ankara University, 06110 Ankara, Turkey; ^3^Faculty of Medicine, Gazi University, 06100 Ankara, Turkey; ^4^Department of Biology, Naples University Federico II, 80134 Naples, Italy

## Abstract

Microscopic patterns of thirty-four urothelial tumors of the urinary bladder of water buffaloes from the Marmara and Black Sea Regions of Turkey are here described. All the animals grazed on lands rich in bracken fern. Histological diagnosis was assessed using morphological parameters recently suggested for the urinary bladder tumors of cattle. Papillary carcinoma was the most common neoplastic lesion (22/34) observed in this study, and low-grade carcinoma was more common (seventeen cases) than high-grade carcinoma (five cases). Papilloma, papillary urothelial neoplasm of low malignant potential (PUNLMP), and invasive carcinomas were less frequently seen. Carcinoma in situ (CIS) was often detected associated with some papillary and invasive carcinomas. De novo (primary) CIS was rare representing 3% of tumors of this series. A peculiar feature of the most urothelial tumors was the presence in the tumor stroma of immune cells anatomically organized in tertiary lymphoid organs (TLOs). Bovine papillomavirus type-2 (PV-2) E5 oncoprotein was detected by molecular and immunohistochemistry procedures. Early protein, E2, and late protein, L1, were also detected by immunohistochemical studies. Morphological and molecular findings show that BPV-2 infection contributes to the development of urothelial bladder carcinogenesis also in water buffaloes.

## 1. Introduction

 Spontaneous tumors of the urinary bladder are very rare in cattle accounting for 0.01% of all bovine malignancies [1]. Conversely, they are common in adult cattle grazing on lands rich in bracken fern [2–4]. This plant contains toxic substances impairing the immune system and carcinogen principles such as ptaquiloside, the prolonged ingestion of which appears to be involved in bladder carcinogenesis. It has been suggested that ptaquiloside causes an increased cell proliferation in bladder urothelium resulting in urothelial dysplasia [5, 6]; furthermore, it is believed that ptaquiloside can act synergistically with bovine papillomavirus type 2 (BPV-2) thus causing bladder tumors in cattle [7]. 

 Papillomavirus infection plays a central role in bladder carcinogenesis of large ruminants [7–11]. In particular, BPV-2 appears to be involved in many urothelial tumors in cattle and water buffaloes [4, 7, 12]. It has been shown that BPV-2 causes in vivo bladder carcinogenesis through the activation of PDGF *β* receptor [12, 13] and/or of Calpain 3 which is responsible for urothelial cell proliferation via E2F3 protein [14]. 

 Tumors of the urinary bladder of buffaloes have sporadically been described [3]. However, BPV-2 infection has just been reported in urothelial tumors as well as in some nonneoplastic lesions of the urinary bladder of buffaloes [12]. 

 The aim of the present paper is to report the microscopic patterns of thirty-four urothelial tumors of the urinary bladder of water buffaloes, twenty-seven of which were associated with papillomavirus infection. All the animals were from the Marmara and Black Sea Regions of Turkey and grazed on pastures contaminated with bracken fern.

## 2. Materials and Methods

 Thirty-four tumor samples of the urinary bladder were collected at public slaughterhouses of Marmara and Black Sea Region (Turkey) (Bafra, Coskun, Bartin) from 3- to 5-year-old castrated male water buffaloes daily grazing on fern-infested lands. Each sample was divided into two halves. One part was fixed in 10% neutral buffered formalin and was processed for paraffin embedding for morphological assessment; the other half was immediately frozen in liquid nitrogen, stored at –80°C until further processed for molecular procedures. 

### 2.1. Histopathology and Immunohistochemistry

Histologic diagnosis of urinary bladder tumors was assessed on 4-*μ*m-thick hematoxylin-eosin- (HE-) stained sections using morphological criteria suggested in a recent report on the new histological classification of urothelial tumors of the urinary bladder of cattle [4]. Immunohistochemistry was performed to detect both early (E) and late (L) papillomavirus proteins. Briefly, the sections were deparaffinized, and then endogenous peroxidase activity was blocked by incubation in 0.3% H_2_O_2_ in methanol for 20 min. Antigen retrieval was performed by pretreating with microwave heating (twice for 5 min each at 750 W) in citrate buffer pH 6.0. The slides were washed with phosphate buffered saline (PBS), pH 7.4, 0.01 M, then incubated for 1 h at room temperature with donkey serum (Sigma-Aldrich, Milan, Italy) diluted at 1 in 10 in PBS. A polyclonal sheep anti-BPV-2 E5 primary antibody (a kind gift from Dr. M. S. Campo, professor Emeritus of Glasgow University, UK) diluted at 1 in 40000 in PBS was applied for 1 h at room temperature in a humid chamber. The sections were rinsed with PBS before application of the donkey anti-sheep biotinylated secondary antibody (Santa Cruz Biotechnology Inc., CA, USA), diluted at 1 in 100 in PBS for 45 min at room temperature. For E2 and L1 detection, the slides were washed three times with PBS, pH 7.4, 0.01 M, then incubated for 1 h at room temperature with protein block serum free (DakoCytomation, Denmark). A polyclonal rabbit anti-BPV-2 E2 primary antibody (a kind gift from Dr. E. Androphy) diluted at 1 in 50 in PBS and a monoclonal mouse anti-HPV-16 L1 (late protein) primary antibody diluted at 1 in 200/300 in PBS were applied overnight at 4°C in a humid chamber. The sections were rinsed three times for 5 min with PBS, incubated for 40 min at room temperature with appropriate biotinylated secondary antibody (labelled streptavidin-biotin (LSAB) Kit; DakoCytomation, Denmark). Finally, all the sections were washed with PBS and then incubated with streptavidin-conjugated to horseradish peroxidase (LSAB Kit; DakoCytomation, Denmark).

 Color development was obtained by treatment with diaminobenzidine (DakoCytomation, Denmark) for 5–20 min. Sections were counterstained with Mayer's hematoxylin. Negative control sections were incubated with PBS instead of primary antibody. 

### 2.2. PCR Reaction

The DNA was extracted from the urinary bladders of water buffaloes using the DNeasy Tissue Kit (Qiagen TM, Germany) according to the manufacturer's instructions.

 For the detection of BPV-2 E5 DNA, specific primers for the E5 ORF (forward primer, 5′-CACTGCCATTTGTTTTTTTC-3′; reverse primer, 5′-GGAGCACTCAAAATGATCCC-3′) were used. Furthermore, specific primers for the L1 region of the BPV-1 (forward primer, 5′-GGCTGAGGACGCTGCTGGTA-3′; reverse primer, 5′-TCTCCGAGCCCCCTCTGGTC-3′) were also employed. Aliquots 50–100 ng of purified DNA were amplified in 25 *μ*L of reaction mixture containing 2 mM MgCl_2_, 200 mM each dNTP, 480 nM of each primer, and 2.5 U of AmpliTaq Gold DNA Polymerase (Applied Biosystems, Monza, Italy). The reaction was carried out in a thermocycler (Veriti, Applied Biosystems) with an initial denaturation step of 3 min. Then, 35 cycles of amplification were carried out with a denaturation step at 95°C for 45 sec, an annealing step at 50°C, 45 sec, for BPV-2 E5, or at 65°C, 40 sec, for BPV-1 L1 and an extension step at 72°C for 1 min. A final extension step at 72°C for 7 min was performed. Detection of the amplified products was carried out by electrophoresis on ethidium bromide-stained agarose gel. In each experiment, a blank sample consisting of reaction mixture without DNA and a positive control composed of BPV-2 clone DNA (a kind gift from Dr. A. Venuti) or of BPV-1 positive sample were included. One *μ*L of the amplified products was subjected to a second run of PCR under the same experimental conditions. Amplified products from the last PCR were electrophoresed in a 2,5% agarose gel and visualized by ethidium bromide stain. 

### 2.3. RNA Extraction

Total RNA was extracted from urinary bladders of water buffaloes using the RNeasy Mini Kit (Qiagen TM, Germany), according to the manufacturer's instructions. The RNA quality was determined by agarose gel electrophoresis and ultraviolet spectrophotometer analysis.

### 2.4. cDNA-E5 Analysis

The reverse transcription reaction contained 4 *μ*L 5x script reaction mix, 1 *μ*L iScript reverse transcriptase, and 500 ng total RNA as the template. The volume was adjusted to 10 *μ*L with RNase free water (Bio-Rad Laboratories, Milan, Italy). The reaction was incubated at 25°C for 5 min, 42°C for 30 min, 85°C for 5 min, and then kept at 4°C for 5 min. The synthesized cDNA was analyzed by PCR with specific primers for the E5 ORF (forward primer, 5′-CACTGCCATTTGTTTTTTTC-3′; reverse primer, 5′-GGAGCACTCAAAATGATCCC-3′). The total reaction volume was 25 *μ*L containing 2.5 *μ*L 10x Gold Buffer, 2 mM MgCl_2_, 200 mM each dNTP, 480 nM of each primer, 2.5 U of AmpliTaq Gold DNA Polymerase (Applied Biosystems, Monza, Italy), and 100 ng reverse transcription products of cDNA as the template. The reaction procedure was as follows: predenaturation at 95°C for 3 min, 35 cycles (denaturation at 95°C for 45 sec, annealing at 50°C for 45 sec, and extension at 72°C for 1 min), and the final extension at 72°C for 7 min. Detection of the amplified products was carried out by electrophoresis on ethidium bromide-stained agarose gel. In each experiment, a blank sample consisting of reaction mixture without DNA and a positive sample consisting of cloned BPV-2 (a kind gift from Dr. A. Venuti) were included.

## 3. Results 

**Figure 1:**
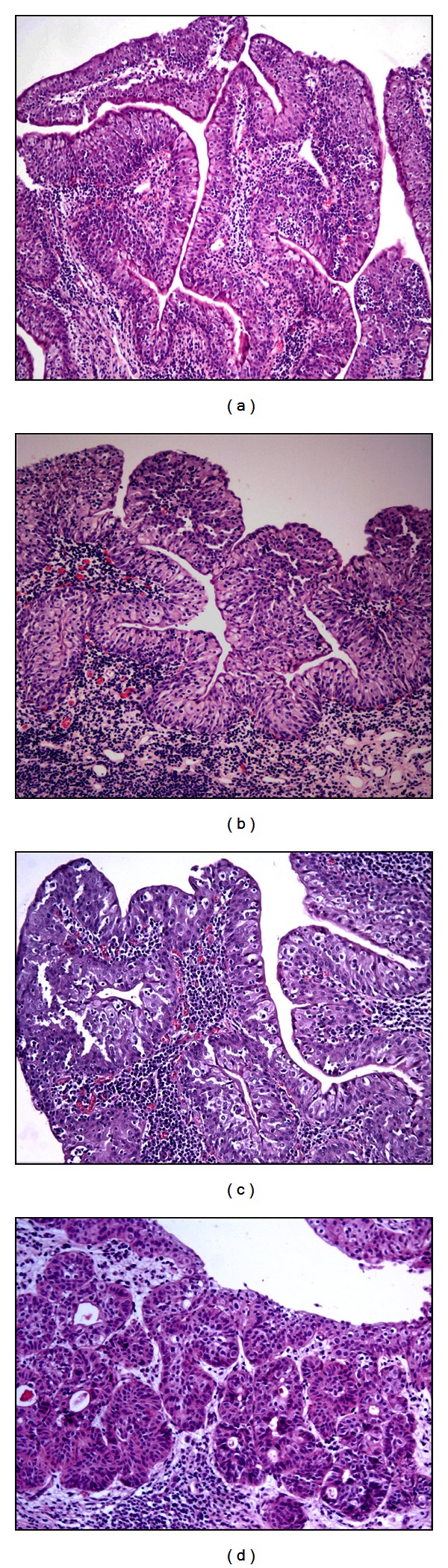
Morphological patterns of the bubaline urothelial tumors. Papilloma (a), H&E, Objective 10x; papillary urothelial neoplasm of low malignant potential (PUNLMP). Notice the diffuse reactive atypia, that is, the presence inflammatory cells into the urothelium (b) H&E, Objective 10x; papillary carcinoma (c) H&E, Objective 20x; invasive carcinoma (d) H&E, Objective 20x.

**Figure 2:**
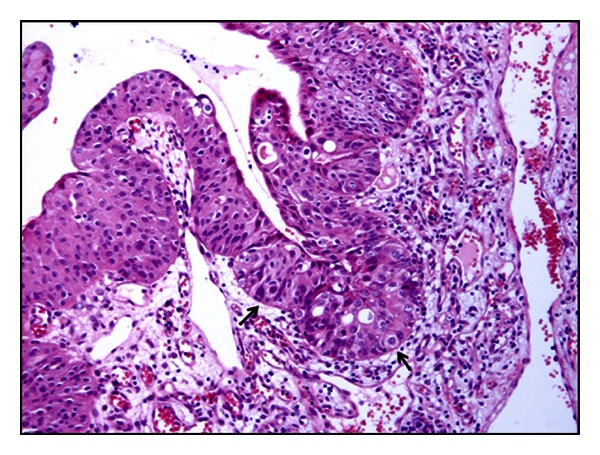
De novo (primary) carcinoma in situ (CIS) H&E, Objective 20x.

 Histological examinations of the urinary bladder tumors detected microscopic patterns consistent with the diagnosis of urothelial papilloma in three cases, two of them showed an exophytic pattern and only one showed an inverted growth. Furthermore, three cases were diagnosed as papillary urothelial neoplasm of low malignant potential (PUNLMP); a diagnosis of papillary carcinoma was performed in twenty-two cases; five cases of invasive carcinomas were also found (Figure 1); de novo (primary) carcinoma in situ (CIS) was seen in only one case (Figure 2).  Table 1 summarizes the results. Tumor-like lesions such as reactive atypia as well as flat and nodular hyperplasia were also seen. 

**Figure 3:**
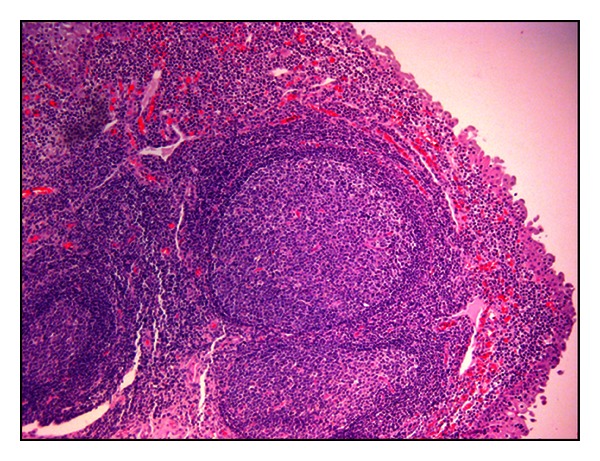
Lymphoid neogenesis. Tertiary lymphoid organs with an evident, clear germinal center and peripheral lymphocytes are evident in tumor stroma. H&E, Objective 10x.

 Papillary carcinoma was the most common neoplastic lesion observed in this study, and low-grade carcinomas were more common (seventeen cases) than high grade carcinomas (five cases). CIS was seen to occur in three buffaloes with papillary carcinoma and in two with invasive bladder neoplasia. They were seen more frequently than the primary (de novo) CIS; the latter was seen in only one case thus representing about 3% of urothelial neoplasms. In all the examined cases, there was a strong inflammation of the tumor stroma; quite frequently, immune cells were anatomically organized in tertiary lymphoid organs (TLOs), in which clear germinal center and peripheral small dark and densely packed mantle lymphocytes without any fibrous encapsulation were seen (Figure 3).

**Figure 4:**
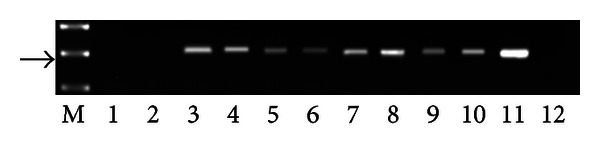
Detection of BPV-2 E5 DNA. M: 50 bp molecular marker (HyperLadder II Bioline); 1-2: urinary bladder samples from healthy buffaloes without BPV-2 E5 DNA; 3–10: some representative bladder tumors samples showing BPV-2 E5 DNA; 11: positive control (cloned BPV-2 DNA); 12: negative control (no DNA added). The arrow indicates the position of the 154 bp BPV-2 E5 PCR product.

**Figure 5:**
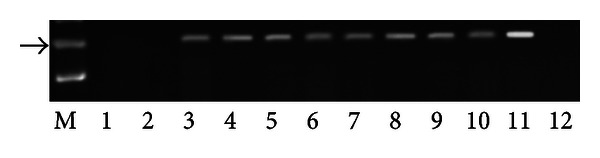
Assessment of BPV-2 E5 cDNA by reverse transcriptase polymerase chain reaction (RT-PCR). M: 50 bp molecular marker (HyperLadder II Bioline); 1-2: healthy urinary bladder samples; 3–10: bladder tumors samples; 11: positive control (cloned BPV-2 DNA); 12: negative control (no DNA added). The arrow indicates the position of the 154 bp BPV-2 E5 PCR product.

**Figure 6:**
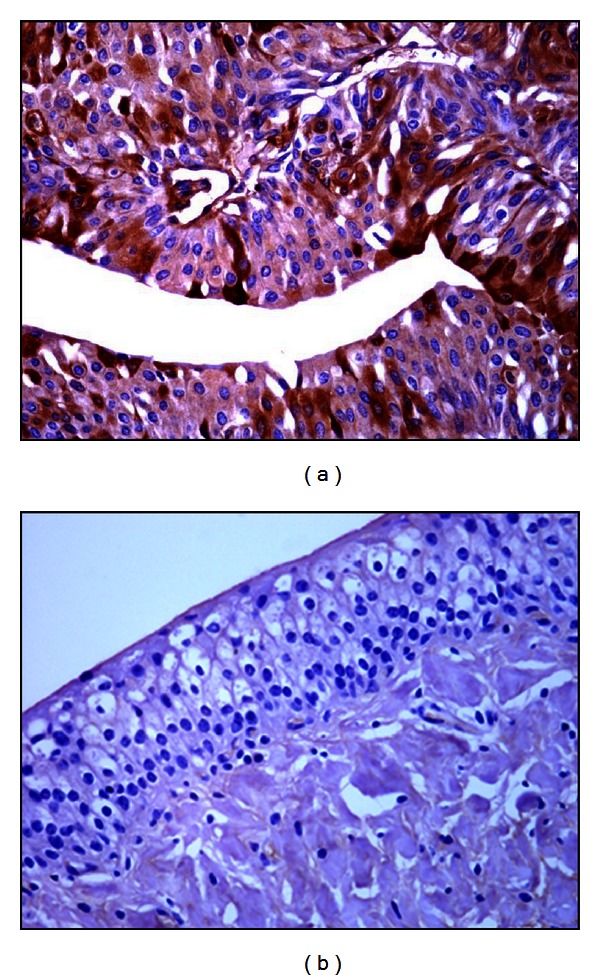
E5 immunohistochemistry. Neoplastic cells show a strong cytoplasmatic E5 immunoreactivity (a), Objective 40x. No E5 immunoreactivity is evident in normal urothelial cells (b), Objective 20x.

**Figure 7:**
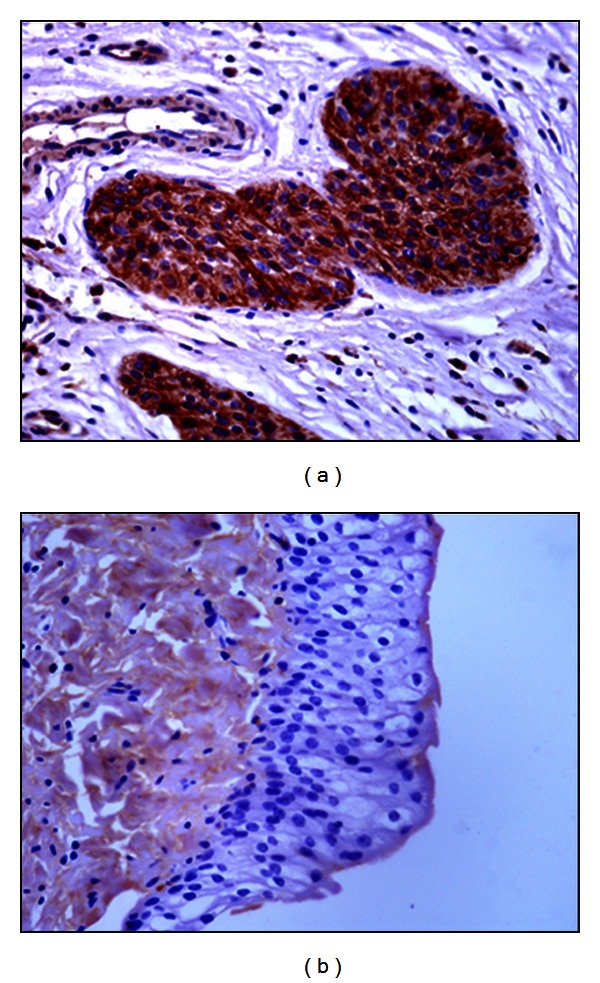
L1 immunohistochemistry. Presence of L1 protein in urothelial neoplastic cells (a) Objective 40x. No L1 protein immunoreactivity was detected in normal urothelial cells (b), Objective 40x.

**Figure 8:**
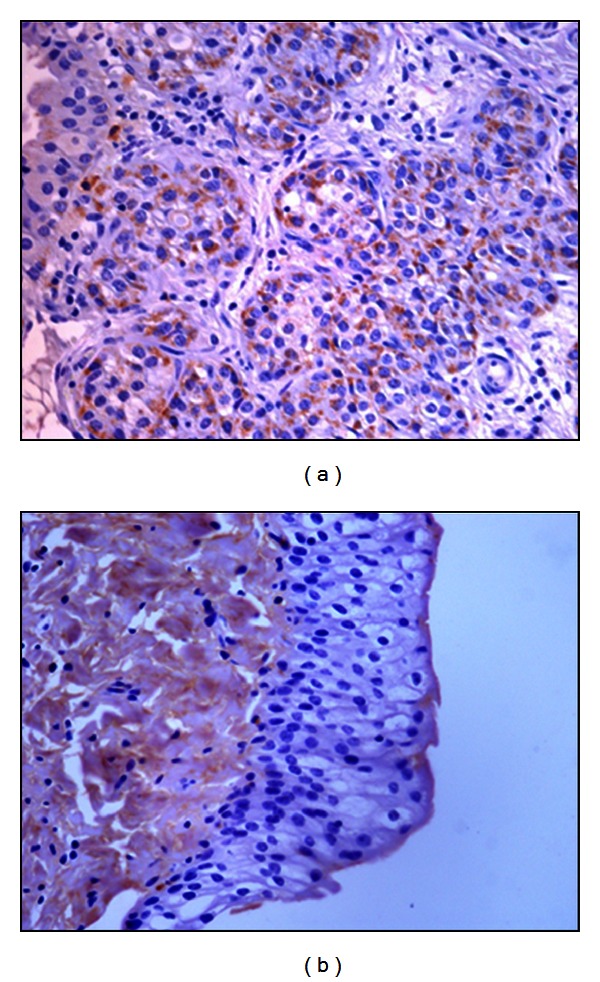
E2 immunohistochemistry. Diffuse immunoreactivity of E2 protein in urothelial neoplastic cells (a), Objective 40x. No E2 protein immunoreactivity was detected in normal urothelial cells (b), Objective 40x.

 PCR analysis demonstrated the presence of E5 DNA in the samples from twenty-seven buffaloes suffering from urothelial bladder tumors in which a fragment of the expected size (154 bp) was amplified (Figure 4). Furthermore, in these samples, reverse transcriptase PCR analysis demonstrated the presence of E5 mRNA (Figure 5). No BPV-1 DNA was detected. Immunohistochemical studies confirmed the presence of E5 protein in the cytoplasm of many neoplastic cells; it was not detected in the cytoplasm of normal cells (Figure 6). Furthermore, a marked immunoreactivity for L1 protein expression was evident both in the cytoplasm and nuclei of the urothelial cells of neoplastic nests; it was not detectable in normal urothelial cells (Figure 7). Since it is known that E2 protein is essential for the viral life cycle and plays a part in productive infection, immunohistochemical studies on the expression of this protein were also carried out. E2 protein expression was detected both in the cytoplasm and nuclei of urothelial cells from neoplastic urinary bladder. Its expression was not manifested in normal urothelial cells (Figure 8).

## 4. Discussion

 The present study reports the microscopic patterns of thirty-four spontaneous urothelial tumors of the urinary bladder in water buffaloes grazed on pasturelands rich in fern. A papillomavirus infection has been shown in twenty-seven tumors (~79%). Both benign and malignant lesions here described fell into the following categories: flat urothelial lesions, papillary urothelial lesions, and invasive urothelial tumors just reported in humans [15] and cattle [4]. No metastases were found in malignant tumors. Our findings corroborate that morphological criteria suggested for histological classification of urothelial tumors in cattle [4] may be used also in water buffaloes. Recently, we have shown that BPV-2 plays a central role also in bubaline bladder carcinogenesis. Like the case in bladder carcinogenesis of cattle, BPV-2 E5 oncoprotein binds to the activated form of platelet derived growth factor *β* receptor (PDGF*β*R) [12]. We document here a papillomavirus infection by western blot analysis, cDNA, and immunohistochemical investigations about the presence of BPV-2 E5 oncoprotein. The expression of BPV-2 L1 protein indicates that a complete life cycle of BPV-2 occurs also in bubaline neoplastic urothelial cells. These findings corroborate our previous results about the crucial role of BPV-2 infection in urothelial cell transformation [12]. Toxic elements of the fern appear to play a synergistic role with BPV-2 infection also in bladder carcinogenesis of water buffaloes. As a matter of fact, when the buffalo breeding based on closed breeding techniques does not allow buffaloes to graze on pastures contaminated with bracken fern, the bladder disease is basically unknown although clinical cases of BPV infection can be seen, that is, papillomatosis of the skin. The microscopical findings of this study show that urothelial tumors of buffaloes share morphological characteristics with the human counterparts just like urothelial tumors of cattle [4, 15, 16]. A peculiar feature of most bubaline urothelial tumors was the presence of diffuse lymphocytes, plasma cells, and macrophages which can evolve into aggregates in the tumor stroma. These findings were also detected in the urothelial cancers of cattle [4], but in buffaloes the immune cells were often organized anatomically in de novo follicles leading to the formation of the so-called tertiary lymphoid organs (TLOs). Chronic follicular cystitis is frequently found in water buffaloes [17]. The pathophysiological significance of the lymphoid neogenesis is still unclear. TLO formation is now recognized as a common feature of many chronic inflammatory diseases and might have a role in maintaining immune responses against persistent antigens. It has been suggested that the stimulus that triggers lymphoid neogenesis in infected tissues is the causative agent itself [18]. It is reasonable to suggest that, in our cases, L1 protein, expressed only in productive BPV-2 infection and known to play a central role both in infection and immunogenicity [19], stimulates adaptive immune responses thus leading to chronic inflammation of tumor stroma in which TLOs can develop. Furthermore, BPV E5 is known to impair the major histocompatibility class I complex (MHC I) thus allowing the persistence of the virus infection. This impairment and immunosuppressant of bracken fern may help maintain antigen stimuli responsible for a heavy cell infiltration which can contribute to TLO formation [7, 20]. It has been suggested that chronic follicular cystitis in buffaloes is characterized by a very strong presence of lymphoid cells [17]. It is worth remembering that the most organized structures are generally found in highly infiltrated tissues [18]. Inflammatory cells of microenvironment and their secreted factors appear to be coresponsible for the fate of a tumor [21] being able to exert a strong anticancer immunosurveillance. Further studies are needed to gather insights into the relationship between tumor cells and their inflammatory microenvironment being a manner of debate [22]. In addition, we have to better understand the role of lymphoid neogenesis in infectious diseases as it has been suggested that, although so far it has been documented in only a few chronic diseases, lymphoid neogenesis might have a protective function by inducing an immune response against the infectious agent. 

## Figures and Tables

**Table 1:** Histological types of thirty-four urothelial tumors of the urinary bladder in water buffaloes.

Microscopic patterns of tumors	Case no.
Papilloma	3
Papillary urothelial neoplasm of low malignant potential (PUNLMP)	3
Low-grade papillary urothelial carcinoma	17
High-grade papillary urothelial carcinoma	5
Low-grade invasive urothelial carcinoma	3
High-grade invasive urothelial carcinoma	2
De novo carcinoma* in situ* (CIS)	1
